# Human gut microbiota adaptation to high-altitude exposure: longitudinal analysis over acute and prolonged periods

**DOI:** 10.1128/spectrum.02916-24

**Published:** 2025-04-21

**Authors:** Xianzong Ma, Changwei Duan, Xiaoying Wang, Yurong Tao, Lang Yang, Yongsheng Teng, Yuanming Pan, Mingjie Zhang, Junfeng Xu, Jianqiu Sheng, Xin Wang, Peng Jin

**Affiliations:** 1 Senior Department of Gastroenterology, The First Medical Center of Chinese PLA General Hospital, Beijing, China; 2 Department of Gastroenterology, The Seventh Medical Center of Chinese PLA General Hospital, Beijing, China; 3 Medical School of Chinese PLA 104607 , Beijing, China; 4 Department of Gastroenterology, Chongqing General Hospital, Chongqing University, Chongqing, China; 5 Cancer Research Center, Beijing Chest Hospital, Capital Medical University, Beijing, China; Central Texas Veterans Health Care System, Temple, Texas, USA

**Keywords:** high altitude, gut microbiota, longitudinal effects, 16S rDNA, dysbacteriosis

## Abstract

This study investigated the longitudinal effects of acute (7-day) and prolonged (3-month) high-altitude exposure on gut microbiota in healthy adult males, addressing the limited data available in human populations. A cohort of 406 healthy adult males was followed, and fecal samples were collected at three time points: baseline at 800 m (406 samples), 7 days after ascending to 4,500 m (406 samples), and 2 weeks post-return to 800 m following 3 months at high altitude (186 samples). High-throughput 16S ribosomal DNA sequencing was employed to analyze microbiota composition and diversity. Results revealed significant changes in alpha- and beta-diversity, with acute high-altitude exposure inducing more pronounced effects compared to prolonged exposure. Specifically, acute exposure increased opportunistic pathogens (*Ruminococcus* and *Oscillibacter*) but decreased beneficial short-chain fatty acid producers (*Faecalibacterium* and *Bifidobacterium*). Notably, these changes in microbiota persisted even after returning to low altitude, indicating long-term remodeling. Functional analyses revealed substantial changes in metabolic pathways, suggesting microbiota-driven adaptations to energy utilization under high-altitude hypoxic conditions. In summary, acute high-altitude exposure caused dramatic changes in gut microbiota, while prolonged exposure led to structural and functional reshaping. These findings enhance our understanding of how high-altitude environments reshape gut microbiota.

**IMPORTANCE:**

This study is the first to investigate the impact of high-altitude exposure on gut microbiota adaptation in a large-scale longitudinal cohort. It seeks to enhance understanding of how high-altitude environments reshape gut microbiota. Acute exposure to high altitude significantly affected both α-diversity and β-diversity of gut microbiota, with acute exposure causing more pronounced changes than prolonged adaptation, indicating temporary disruptions in microbial communities. Notable shifts in microbial abundance were observed, including increased levels of genera linked to hypoxic stress (e.g., *Gemmiger*, *Ruminococcus*, and *Parabacteroides*) and decreased levels of beneficial bacteria (e.g., *Faecalibacterium*, *Roseburia*, and *Bifidobacterium*), suggesting possible adverse health effects. Functional analysis indicated changes in metabolism-related pathways post-exposure, supporting the idea that high-altitude adaptations involve metabolic adjustments for energy management. These findings enhance understanding of high-altitude physiology, illustrating the role of gut microbiota in hypoxic health.

## INTRODUCTION

High altitude in medicine refers to an area over 2,500 m that remarkably affects the functionality of humans, and its environmental characteristics include low partial pressure of oxygen, dramatic climate, and intense ultraviolet radiation (1). Upon entering high-altitude regions, these factors trigger a range of physiological responses, ultimately leading to altitude-related illnesses (2). Specifically, acute exposure to low-pressure hypoxia can cause the onset of various acute altitude illnesses, including acute altitude sickness, acute cerebral edema, and acute pulmonary edema (3–5). Moreover, prolonged exposure led to the occurrence of chronic altitude illnesses, such as altitude hypertension, pulmonary artery hypertension, and plateau heart disease, which can be life-threatening for people in high-altitude regions (5, 6). Published studies reported that high-altitude stimulus factors, such as hypoxia and low temperature, could profoundly alter the composition and structure of gut microbiota of human beings (7–9).

The homeostasis of gut microbiota has emerged as a critical modulator of human health, influencing a wide range of physiological processes, including mucosal immune regulation, maintenance of barrier integrity, metabolic homeostasis, energy production, and psychological well-being through the flora–blood–hepatic and adipose axes, production of short-chain fatty acids (SCFAs), and metabolism of bile acids, etc. (9–12). An imbalance in gut microbiota could disturb these critical processes and is extensively associated with gastrointestinal disorders, autoimmune illnesses, metabolic diseases, and mental conditions, such as anxiety and depression (13–18). Suzuki et al. (19) found that altitude was positive correlated with the abundance of anaerobic bacteria but negatively correlated with that of facultative anaerobic, microaerobic, and aerobic bacteria in wild house mice, suggesting that hypoxia shaped gut flora. Additionally, Karl et al. (20) analyzed the gut microbiota from a small cohort of healthy adult males at sea level and high altitude (4,300 m) and the relationship between the gut microbiota and the hosts’ response to altitude. They found that altitude-associated structural changes in the gut microbiota might contribute to the development of acute mountain sickness. More results from animal models exposed to high-altitude hypoxia showed that hypoxia induced dysbiosis, such as increases in genus *Parabacteroides*, *Alistipes*, *Lactococcus*, *Bacteroides*, *Prevotella* and decreases in genus *Helicobacter, Faecalibaculum*, and *Bifidobacterium*, which was associated with weight loss, erythrocytosis, cardiac hypertrophy, and changes in energy metabolism and carbohydrate digestion (7, 21–23). Current research demonstrates that a high-altitude environment induces structural and functional modifications in gut microbiota. In extreme cases, these perturbations may progress to microbial dysbiosis, a pathogenic mechanism implicated in the initiation and exacerbation of altitude-associated pathologies. Therefore, to prevent the occurrence of high-altitude events, it is crucial to understand how acute and prolonged high-altitude exposure affects the gut microbiota of humans. However, studies simulating high-altitude exposure using animal models dominate this field (19, 22–25). Data are limited on how high-plateau environment impacts the gut microbiota of hosts, particularly from large-scale, longitudinal human studies. Clarifying this relationship could provide valuable insights into preventing and managing altitude illnesses among acclimatized populations.

To provide more robust evidence, our study is the first to utilize high-throughput 16S rDNA Illumina HiSeq sequencing (V3–V4 region) to investigate the acute (7-day) and prolonged (3-month) effects of high-altitude exposure on gut microbiota in a large, longitudinally tracked human cohort.

## MATERIALS AND METHODS

### Participants and samples

This longitudinal prospective observational study recruited participants from low-altitude regions (Urumqi, Xinjiang Uygur Autonomous Region, China; 800 m) who were required to work at high altitudes (Hotan County, Hotan Prefecture, Xinjiang Uygur Autonomous Region, China; 4,500 m) for 3 months before returning to Urumqi. Participants aged 18 years or older were eligible for inclusion after providing informed consent. Exclusion criteria included antibiotic or probiotic use within the past 3 months, a history of chronic gastrointestinal or metabolic diseases, or recent high-altitude exposure. Baseline demographic and lifestyle data, including age, height, weight, smoking history, tea consumption, and alcohol use, were collected. To minimize confounding factors, all participants adhered to a supervised daily regimen of 8-hour physical activity (e.g., altitude acclimatization tasks) with rest intervals for safety, alongside a standardized diet (55%–65% carbohydrates, 20%–30% fats, and 12%–15% proteins) adjusted to individual metabolic needs (3,000–3,500 kcal/day) based on plateau labor intensity.

Fecal samples were collected at three time points from the same cohort. Group I (G-I) samples were collected at the baseline altitude (800 m, Urumqi). Group II (G-II) samples were taken 7 days after participants ascended to 4,500 m (Hotan County). Group III (G-III) samples were collected 2 weeks after their return to 800 m (Urumqi) following a 3-month stay at 4,500 m. Samples were preserved in dry ice during collection, transported under controlled conditions (cold-chain transportation, temperature below −60°C), and stored at −80°C in the Department of Gastroenterology, Seventh Medical Center of Chinese PLA General Hospital until analysis. The study was approved by the institutional ethics committee of the Seventh Medical Center of Chinese PLA General Hospital. Sequencing data have been uploaded to GenBank (BioProject: PRJNA1194026).

### DNA extraction and sequencing

Bacterial DNA extraction from thawed fecal samples was performed using the QIAamp DNA stool minikit as per the manufacturer’s instructions without modifications. After extraction, the quality of the extracted DNA was checked with Thermo NanoDrop 2000 and 1% agarose gel. The V3–V4 region of the 16S rDNA gene from the fecal DNA was amplified using the barcoded primers 341F (CCTACGGGRSGCAGCAG) and 806R (GGACTACVVGGGTATCTAATC). The amplified products were purified using the AxyPrep DNA Gel Extraction Kit (Axygen Biosciences, Union City, CA, USA). DNA libraries were constructed and sequenced on the Illumina NovaSeq PE250 platform by Realbio Technology (Shanghai, China).

PANDAseq (version 2.9) was used to merge paired-end reads, and clean reads were obtained after the removal of low-quality reads (with a quality value < 20, containing more than three nitrogenous bases or length ranging from 250 to 500 nt). Usearch (version 7.0.1090) was used to cluster the operational taxonomic units (OTUs) with a 97% sequence similarity (26). However, in this study, OTUs were chosen to maintain methodological consistency with previous studies in high-altitude or hypoxic environments, facilitating comparisons across similar data sets (19, 20). The most abundant reads from each OTU cluster were taxonomically identified using RDP classifier; only annotations with ≥80% confidence levels were accepted (27).

### Data processing and analysis

In terms of participants’ characteristics, continuous data are expressed as the mean ± standard deviation (SD). Categorical data are represented by the number of cases and percentages. Continuous variables were analyzed using ANOVA; categorical variables were analyzed using chi-square tests. These analyses were performed using the SPSS 26.0 software (IBM Corporation, Armonk, NY, USA). *P* < 0.05 indicates a statistically significant difference.

In terms of gut microbiota, an independent sample *t*-test was used to analyze normally distributed variables. Non-normally distributed variables were analyzed using the Mann–Whitney *U* test. The alpha-diversity index analysis was performed using QIIME 1 software (QIIME version 1.9.1). Each index of α-diversity was analyzed by the rank sum test. If two sets of samples are compared, the wilcox.test function in R is used, and if more than two sets of samples are compared, the kruskal.test function in R is used. Analysis of similarities was performed using the vegan package in R. Principal coordinates analysis (PCoA) was performed using the ade4 package in R. Permutational multivariate analysis of variance (Adonis analysis) was implemented in the vegan package of R language. Linear discriminant analysis (LDA) effect size (LEfSe) was performed using the LEfSe Tools. Phylogenetic examination of communities was achieved via the reconstruction of unobserved states (PICRUSt) analysis using the PICRUSt software. The LEfSe analysis method was used to estimate the impact of each component’s metabolic pathway on the difference effect and to identify the metabolic pathway that had a significant difference effect on sample division (the default screening condition was LDA ≥ 2). The Spearman correlation heatmap between species and environmental factors was plotted using the corrplot package of R software.

## RESULTS

### Participants’ characteristics and sequencing information

**Fig 1:**
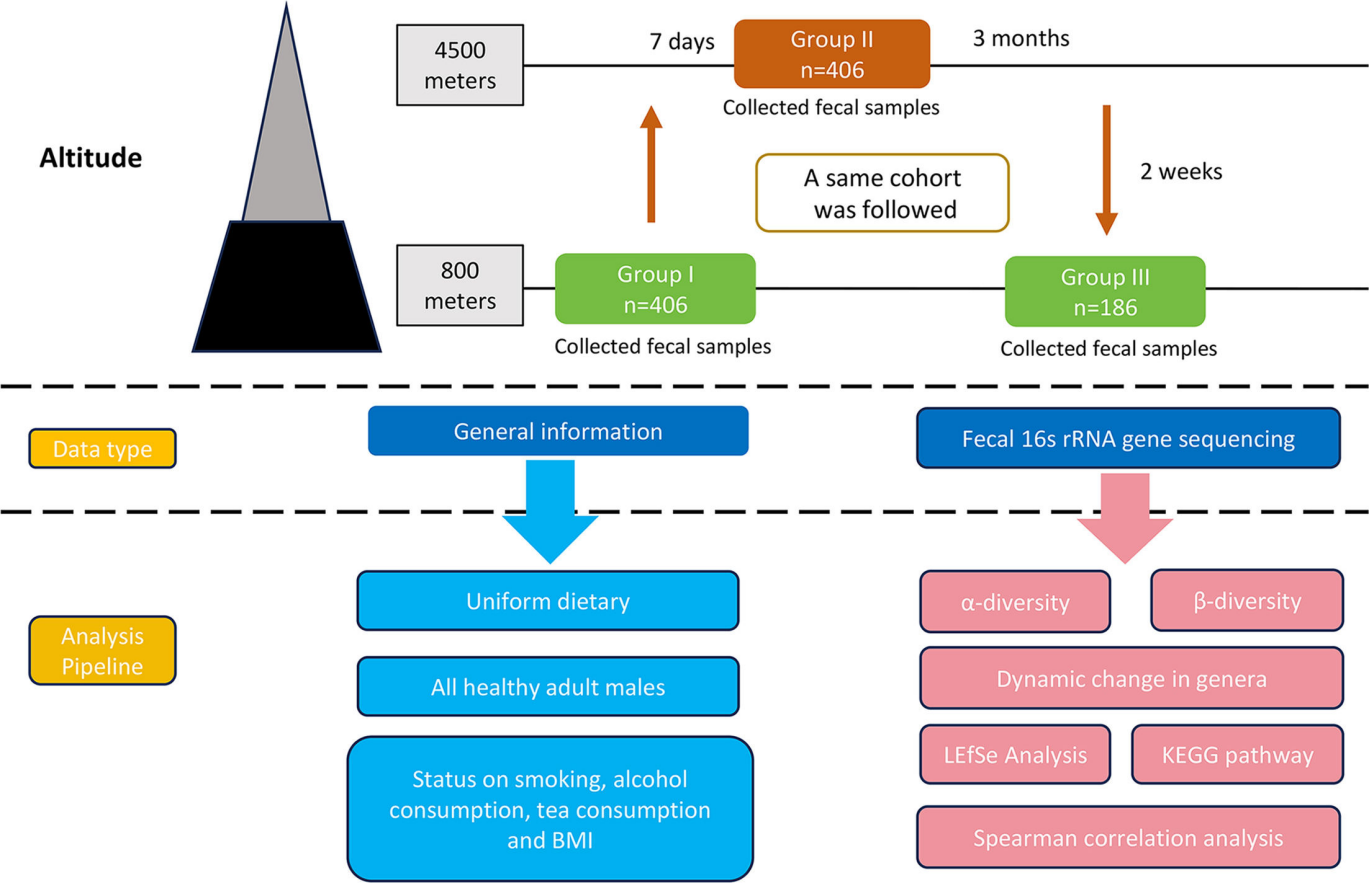
Overview of the analysis pipeline. The fecal and baseline characteristics were collected and analyzed from populations at different altitudes. The fecal samples were subjected to the 16S rRNA gene sequencing followed by diversity analysis, bacterial composition analysis, linear differential analysis, and KEGG functional pathway.

In total, 998 fecal samples were collected from 406 healthy adult males: 406 samples from Group I, 406 samples from Group II, and 186 samples from Group III, with the reduced sample size due to various reasons, such as participants being on leave during the final collection period (Fig. 1). The baseline characteristics of all participants are presented in Table 1. The mean age of participants in Groups I, II, and III was 22.63, 22.65, and 22.19 years, respectively. There were no significant differences in age distribution, body mass index (BMI), smoking status, drinking habits, or tea consumption among the three time points (*P* > 0.05). A total of 224,044 OTUs were identified across all samples, with group-specific OTU counts of 1,434, 1,577, and 1,203 for Groups I, II, and III, respectively (Fig. S1).

**TABLE 1:** The profiling of participants (all healthy adult males)^*a*^

Indicators	Group I^*b*^ (*N* = 406)	Group II^*c*^ (*N* = 406)	Group III^*d*^ (*N* = 186)	*P*-value
Years (mean ± SD)	22.63 ± 3.04	22.65 ± 3.07	22.19 ± 2.71	0.187
BMI (mean ± SD)	22.19 ± 2.12	22.14 ± 2.15	22.10 ± 2.01	0.899
Smoking				0.350
Yes	249 (61.3)	271 (61.9)	104 (55.9)	
No	157 (38.7)	167(38.1)	82 (44.1)	
Alcohol consumption				0.582
Yes	11 (2.7)	8 (1.8)	3 (1.6)	
No	395 (97.3)	430 (98.2)	183 (98.4)	
Tea consumption				0.848
Yes	203 (50.0)	212 (48.4)	94 (50.5)	
No	203 (50.0)	226 (51.6)	92 (49.5)	

^*a*^ Annotation: a cohort of 406 healthy adult males was followed at three time points.

^*b*^ Baseline at 800 m (406 samples).

^*c*^ Seven days after ascending to 4,500 m (406 samples).

^*d*^ Two weeks post-return to 800 m following 3 months at high altitude (186 samples).

### High-altitude exposure significantly affected the α-diversity and β-diversity of gut microbiota

**Fig 2:**
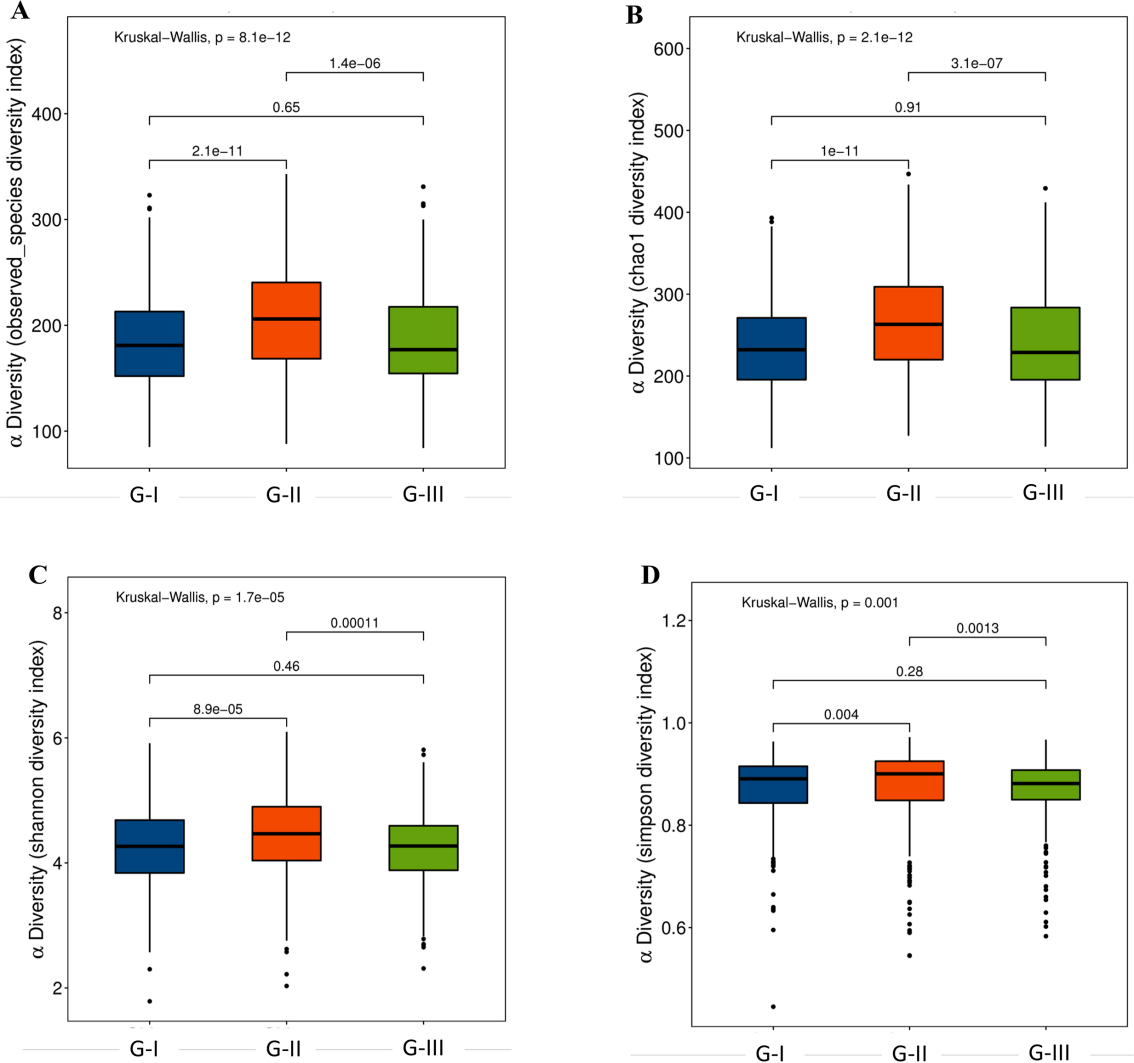
Alpha-diversity index box plot. The community richness between G-I, G-II, and G-III is shown in A (observed species diversity index) and B (Chao1 index), and the species diversity of microbiota is shown in C (Shannon diversity) and D (Simpson diversity index). The horizontal axis represents the sample grouping, and the vertical axis represents the α-diversity index value of different groups.

To investigate the adaptive responses of gut microbiota to acute and prolonged high-altitude exposure, we first analyzed the α-diversity, including community richness (observed species and chao1) and diversity (Shannon and Simpson indices). As shown in Fig. 2, there were significant differences in the α-diversity between G-I and G-II, G-II and G-III (*P* < 0.05). Notably, α-diversity in G-III reverted to levels indistinguishable from G-I (*P* > 0.05), suggesting that the α-diversity changes were transient and dependent on the persistence of high-altitude conditions.

**Fig 3:**
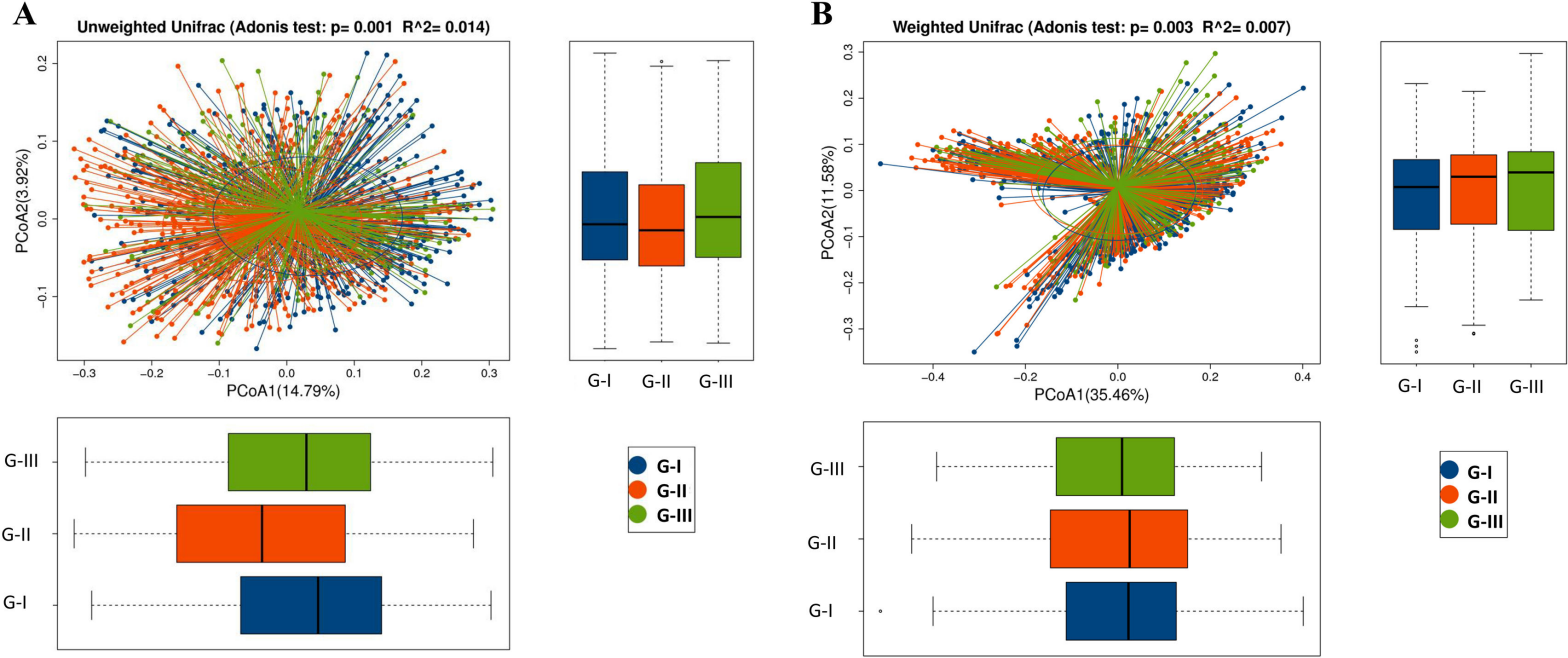
Microbial community structure between three groups. (A) Unweighted UniFrac PCoA. (B) PCoA of weighted UniFrac. The percentage represents the contribution rate of the principal dimension to the sample difference.

To understand the structural changes in the gut microbiota, we performed PCoA with Adonis tests using both unweighted (species presence/absence) and weighted (species abundance) UniFrac distances (Fig. 3; Fig. S2). Both acute and prolonged high-altitude exposure (G-II and G-III) caused significant β-diversity shifts compared to G-I. Critically, these β-diversity alterations persisted even after returning to 800 m (G-I vs G-II vs G-III: Adonis test: Unweighted UniFrac, *P* = 0.001, *R*^2^ = 0.014; Weighted UniFrac, *P* = 0.003, *R*^2^ = 0.007. G-I vs G-II: Adonis test: Unweighted UniFrac, *P* = 0.001, *R*^2^ = 0.013; Weighted UniFrac, *P* = 0.001, *R*^2^ = 0.008. G-I vs G-III: Adonis test: Unweighted UniFrac, *P* < 0.001, *R*^2^ = 0.007; Weighted UniFrac, *P* = 0.034, *R*^2^ = 0.006. G-II vs G-III: Adonis test: Unweighted UniFrac, *P* = 0.001, *R*^2^ = 0.009; Weighted UniFrac, *P* = 0.002, *R*^2^ = 0.008), indicating that high-altitude exposure induced long-lasting reorganization of gut microbial community structure, independent of α-diversity recovery.

### High-altitude exposure modified the relative abundance of gut microbiota

**Fig 4:**
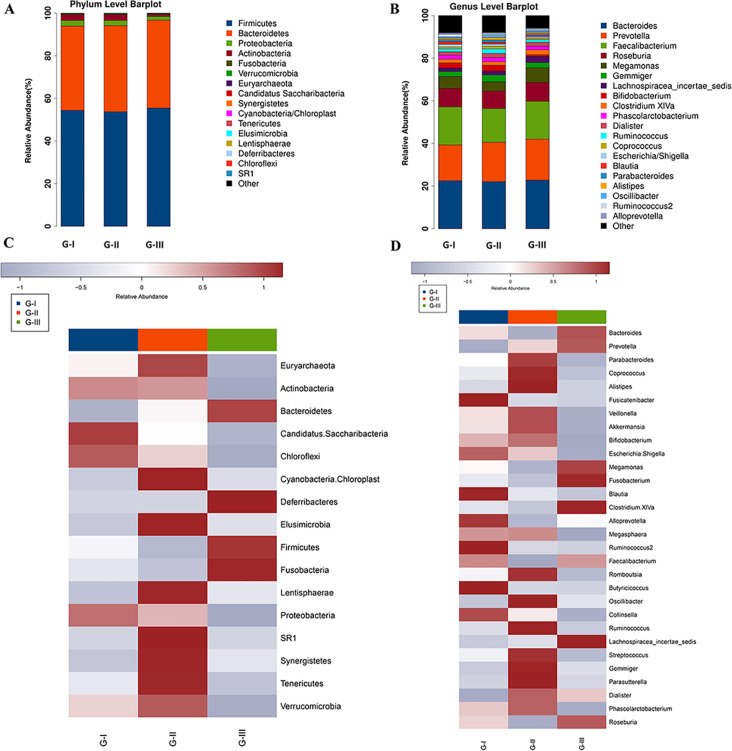
Species classification and abundance analysis of three groups. (A) The phylum bar graph of the intestinal microbiota of each group. (B) Heatmap clustering analysis at the phylum classification level. *Z*-score normalization is now performed horizontally across the three groups for each phylum/genus, with group averages calculated prior to normalization. The use of color gradients clearly illustrates the different enrichment patterns (highest values are red, and lowest values are purple). (C) The genus bar graph of the intestinal microbiota of each group. (D) Heatmap clustering analysis at the genus classification level.

To further analyze whether the high altitude had a longitudinal effect on the composition of gut microbiota, we compared the variation in the classification and relative abundance of the microbiota at each time point at different taxonomic levels (Fig. 4). At the phylum level, Firmicutes and Bacteroidetes were the first and second in relative abundance among all groups, followed by Proteobacteria, Actinobacteria, Fusobacteria, and Verrucomicrobia. The relative abundance of Bacteroidetes showed an increasing trend in G-I, G-II, and G-III, while the relative abundance of Proteobacteria and Actinobacteria showed a downward trend, especially in G-III (Fig. 4A). In the plateau (G-II), the relative abundance of Verrucomicrobia and Euryarchaeota increased, while that of Fusobacteria and Firmicutes decreased (Fig. 4B). At the genus level, *Bacteroides*, *Prevotella*, *Faecalibacterium*, *Roseburia*, *Megamonas*, *Gemmiger*, *Bifidobacterium*, *Lachnospiraceae_incertae_sedis*, *Phascolarctobacterium*, and *Clostridium XIVa* were the top 10 genera in relative abundance (Fig. 4C). The relative abundance of *Bacteroides*, *Faecalibacterium, Roseburia*, *Megamonas*, and *Clostridium XIVa* decreased in G-II compared to G-I and G-III, whereas *Gemmiger*, *Bifidobacterium,* and *Phascolarctobacterium* increased in relative abundance. The relative abundance of *Prevotella* and *Lachnospiraceae_incertae_sedis* incrementally increased over the three time points (Fig. 4D), suggesting that the high-altitude exposure might have a sustained impact on the structure and relative abundance of gut microbiota.

### Unique microflora characteristics after acute and prolonged exposure at high altitude

**Fig 5:**
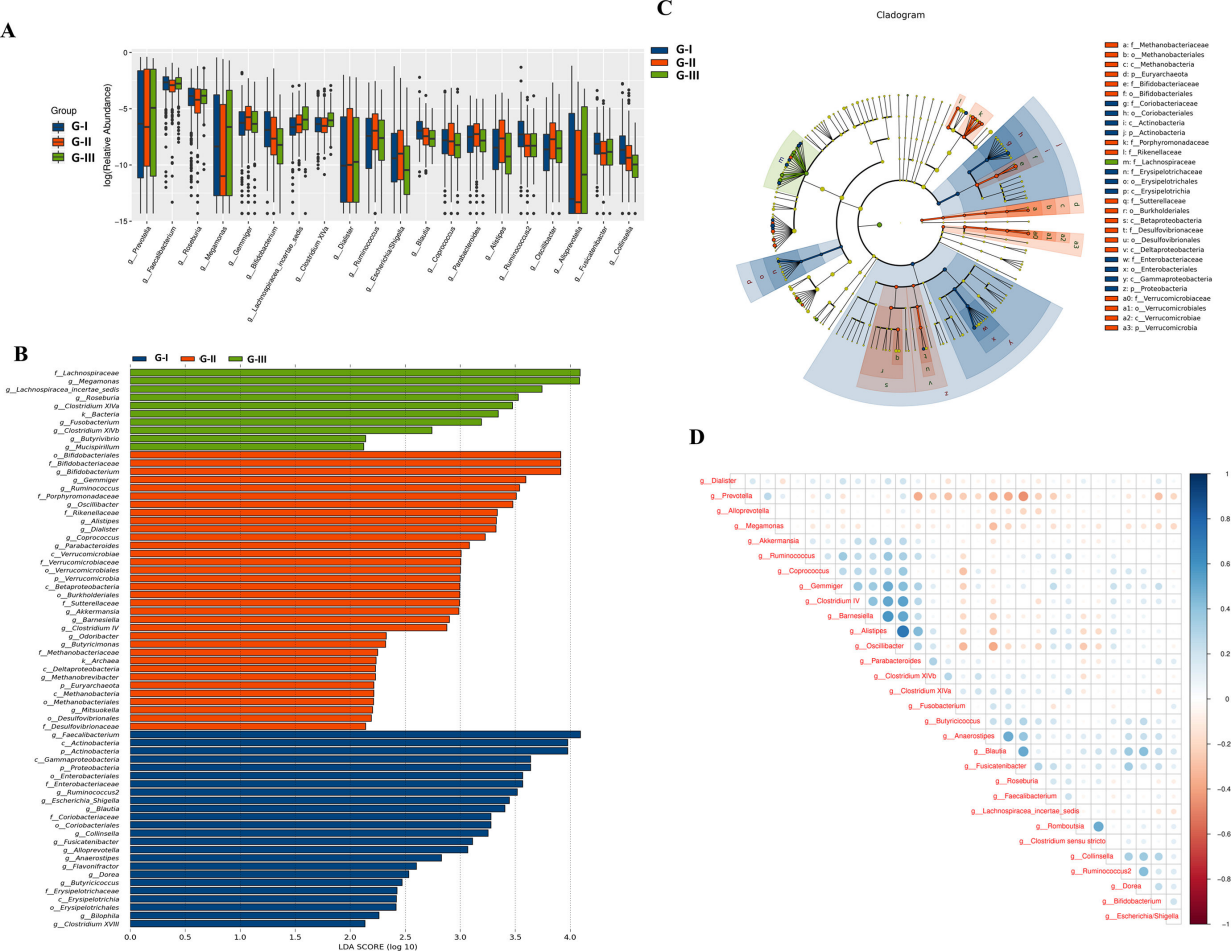
Significant difference analysis of gut microbiota of three groups. (A) Rank sum test analysis between groups (*P* < 0.05). (B and C) LEfSe analysis. The figure lists bacterial communities with LDA score (log 10 > 2) and *P* < 0.05. p, phylum; c, class; o, order; f, family; and g, genus. (D) Spearman correlation analysis of genera with significant differences among groups.

In the following, we further analyzed the characteristics of gut microbiota at different altitudes and times. As presented in Fig. 5A, at the genus level, the top 20 species that were significantly different were *Prevotella*, *Faecalibacterium*, *Roseburia*, *Gemmiger*, *Megamonas*, *Bifidobacterium*, *Lachnospiracea_incertae_sedis*, *ClostridiumXIVa*, *Dialister*, *Ruminococcus*, *Escherichia/Shigella*, *Blautia*, *Coprococcus*, *Parabacteroides*, *Alistipes*, *Ruminococcus_2*, *Oscillibacter*, *Alloprevotella*, *Fusicatenibacter*, and *Collinsella*. Among them, the genera that significantly increased in G-II compared to G-I and G-III were *Gemmiger*, *Ruminococcus*, *Escherichia/Shigella*, *Parabacteroides*, *Alistipes*, and *Oscillibacter*, while the genera with significant decreases were *Faecalibacterium*, *Roseburia*, *Megamonas*, and *Alloprevotella*. Under three consecutive time points, the genera *Bifidobacterium*, *Blautia,* and *Collinsella* continued to decrease significantly, while *Lachnospiraceae_incertae_sedis* and *Dialister* continued to increase significantly.

Subsequently, on the basis of the identified significantly different species among groups, we found that the characteristic genera of G-I were *Faecalibacterium*, *Ruminococcus_2*, *Escherichia_Shigella*, *Blautia*, *Collinsella*, *Fusicatenibacter*, *Alloprevotella*, *Anaerostipes*, *Flavonifractor*, *Dorea*, *Butyricicoccus*, *Bilophila,* and *Clostridium XVIII;* the characteristic genera of G-II were *Bifidobacterium*, *Gemmiger*, *Ruminococcus*, *Oscillibacter*, *Alistipes*, *Dialister*, *Coprococcus*, *Parabacteroides*, *Akkermansia*, *Barnesiella*, *Clostridium IV*, *Odoribacter*, *Butyricimonas, Methanobacteriaceae*, and *Mitsuokella;* the characteristic genera of G-III were *Megamonas*, *Lachnospiraceae_incertae_sedis*, *Roseburia*, *Clostridium XIVa*, *Fusobacterium*, *Clostridium XIVb*‚ *Butyrivibrio*, and *Mucispirillum* (Fig. 5B and C; LDA score: log 10 > 2). Figure 5D further elucidates the unique patterns or relationships among the differential microbiota, revealing positive correlations among *Alistipes*, *Gemmiger*, *Oscillibacter*, *Coprococcus*, and *Clostridium IV*.

### The functional changes in gut microbiota after acute and prolonged high-altitude exposure had unique characteristics

**Fig 6:**
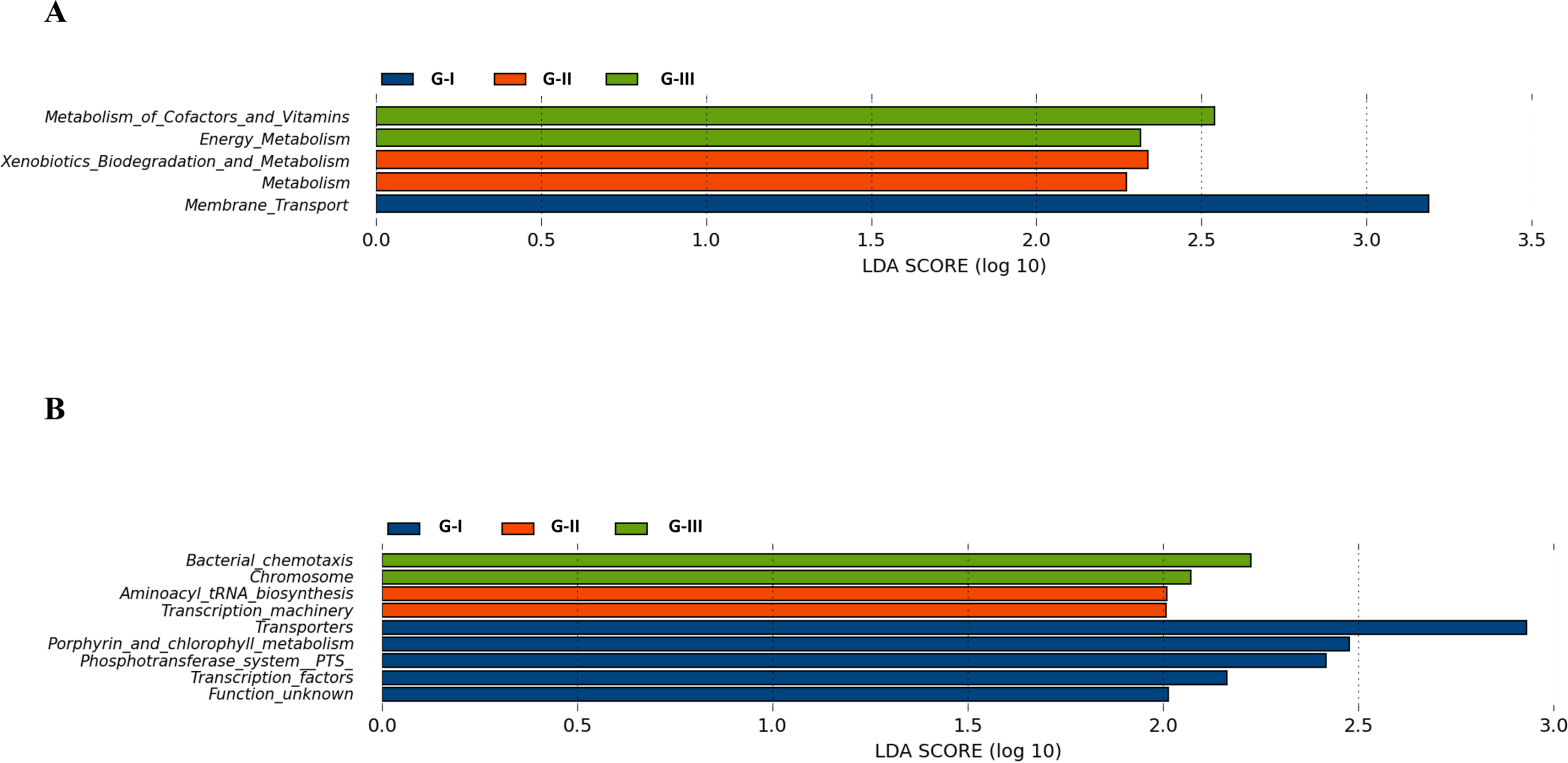
Microbiota functional analysis of three groups. (A) The enriched Kyoto Encyclopedia of Genes and Genomes database pathway of level 2. (B) The enriched KEGG pathway of level 3.

We analyzed the Kyoto Encyclopedia of Genes and Genomes database (KEGG) pathways of intestinal flora at different altitudes by the PICRUSt2 method (28). The results revealed that there were five and nine functional pathways with significant differences in KEGG pathways at level 2 (subcategory of metabolism and biological pathways) and level 3 (specific pathways within subcategories), respectively (Fig. 6). At level 2, the microbiota function of G-I was significantly enriched in membrane translocation; the microbiota functions of G-II were significantly enriched in xenobiotic biodegradation and metabolic pathways, while those of G-III were significantly enriched in energy metabolism and metabolism of cofactors and vitamins (Fig. 6A). At level 3, the microbiota functions of G-I were significantly enriched in transporters, porphyrin and chlorophyll metabolism, the phosphotransferase system, and transcription factors. The microbiota functions in G-II were significantly enriched in Aminoacyl tRNA biosynthesis (which is related to protein metabolism) and transcription machinery and that of G-III were significantly enriched in Bacterial chemotaxis and Chromosome (Fig. 6B).

### Correlation analysis between lifestyle factors and gut bacterial genera

**Fig 7:**
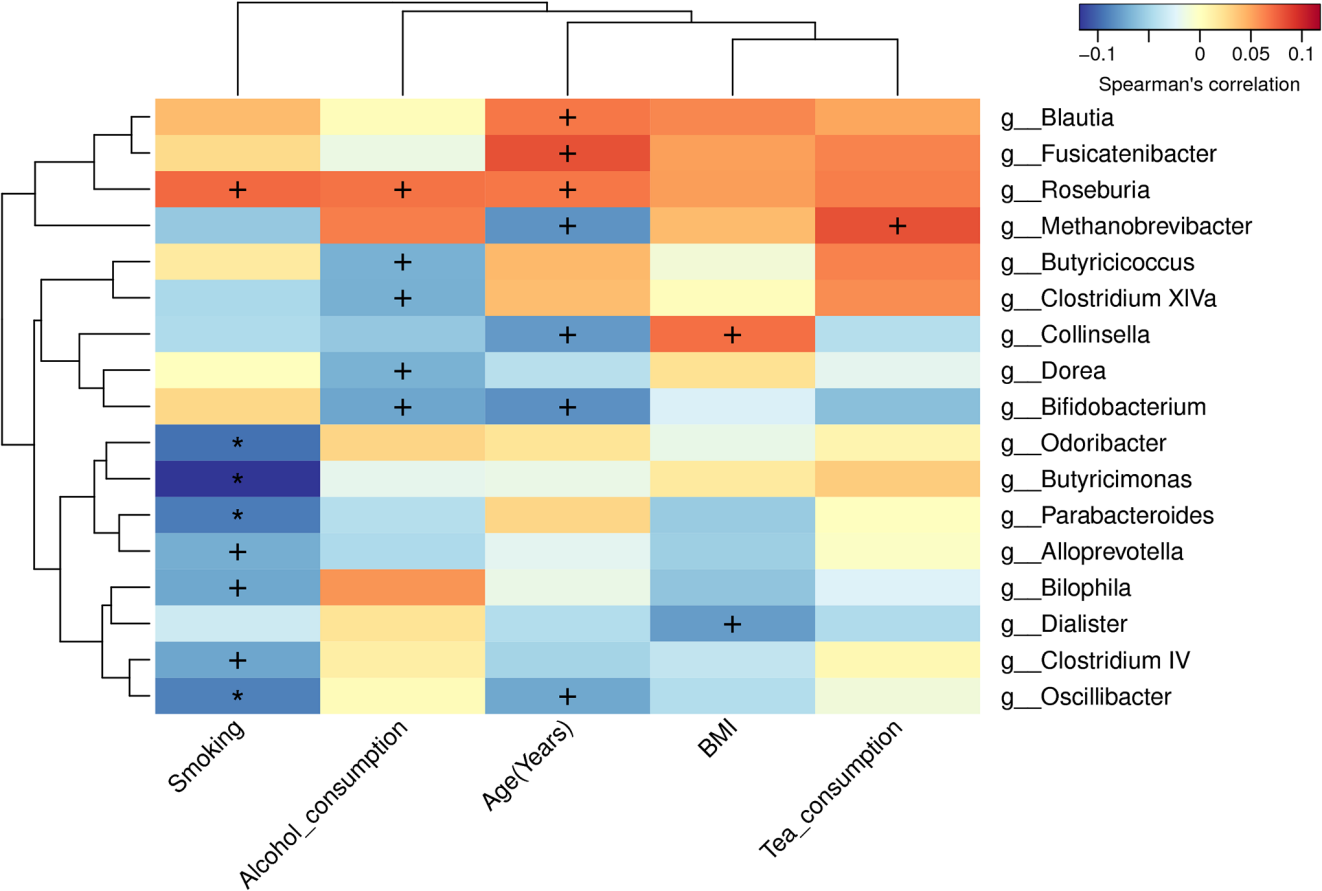
Spearman correlation analysis of genera with lifestyle factors. *^+^P* < 0.05 and *P < 0.01.

The Spearman correlation analysis was conducted to investigate the associations between lifestyle factors, such as smoking, alcohol consumption, age, body mass index, and tea consumption, and various characteristic bacterial genera (Fig. 7). The results showed that age exhibited a significantly negative correlation with the abundance of *Bifidobacterium* (*R* = −0.082, *P* = 0.013), *Collinsella* (*R* = −0.078, *P* = 0.018), *Methanobrevibacter* (*R* = −0.081, *P* = 0.014), and Oscillibacter (*R* = −0.072, *P* = 0.030), but positively correlated with that of *Roseburia* (*R* = 0.067, *P* = 0.045)*, Fusicatenibacter* (*R* = 0.081, *P* = 0.015), and *Blautia* (*R* = 0.067, *P* = 0.044). BMI showed a negative correlation with *Dialister* (*R* = −0.077, *P* = 0.019) but a positive correlation with *Collinsella* (*R* = 0.069, *P* = 0.036). Smoking was significantly negatively correlated with bacterial genera, such as *Odoribacter* (*R* = −0.095, *P* = 0.004), *Butyricimonas* (*R* = −0.117, *P* < 0.001)*, Parabacteroides* (*R* = −0.090, *P* = 0.006), *Oscillibacter* (*R* = −0.089, *P* = 0.007), *Bilophila* (*R* = −0.072, *P* = 0.029), *Alloprevotella* (*R* = −0.069, *P* = 0.036), and *Clostridium IV* (*R* = −0.073, *P* = 0.027)‚ but positively correlated with *Roseburia* (*R* = 0.072, *P* = 0.030). Alcohol consumption was negatively correlated with *Bifidobacterium* (*R* = −0.073, *P* = 0.027), *Dorea* (*R* = −0.067, *P* = 0.041), *Clostridium XIVa* (*R* = −0.069, *P* = 0.039), and *Butyricicoccus* (*R* = −0.068, *P* = 0.040) but positively correlated with *Roseburia* (*R* = 0.068, *P* = 0.040). Additionally, the habit of tea consumption positively correlated with *Methanobrevibacter* (*R* = 0.081, *P* = 0.015).

## DISCUSSION

The human gut microbiota has been found to undergo dynamic changes in response to high-altitude stressors. Yet, the longitudinal patterns of these adaptations and their functional consequences remain poorly understood. Our study, utilizing a rigorously controlled cohort of 406 healthy males across three altitude-exposure phases, yielded three key findings: acute hypoxia triggered transient α-diversity fluctuations but induced persistent β-diversity restructuring, even after returning to low altitude; hypoxia reshaped microbial ecology through opposing trends in opportunistic pathogens vs functionally critical SCFA producers; and these structural changes drove altitude-specific metabolic reprogramming, with implications for energy harvesting and mucosal immunity.

High-altitude hypoxia induced microbial restructuring, accompanied by diversity dynamics, and led to taxonomic shifts. The rapid increase in α-diversity during acute exposure aligned with previous findings from rodent studies, where the α-diversity of the gut microbiota showed a significant increase in rates in pikas acutely exposed to high-altitude hypoxia, reflecting the plasticity of the gut microbiota and demonstrating its rapid response and adaptability to the environment (7, 29). However, the full α-diversity recovery post-repatriation contrasts with animal models (22), suggesting human-specific resilience. Conversely, β-diversity shifts persisted across all phases, indicating irreversible community reorganization. Previous studies have shown that the gut microbiota of rhesus monkeys living at high altitudes (such as in Tibet) exhibits a unique abundance and structural composition, distinct from that of other rhesus monkeys, despite sharing core flora (30). Furthermore, Zhang et al. (23) revealed a marked divergence in the β-diversity of gut microbiota among mice inhabiting distinct altitudes. These findings imply that while species richness rebounds, hypoxia alters interspecies relationships, underscoring the profound dynamic interplay between high-altitude environmental pressure and microbial community adaptation. These alterations are further confirmed by difference analysis, showing unique biomarker taxa for each phase. Notably, acute altitude advancement might cause a decrease in the ratio of Firmicutes/Bacteroidetes (F/B), and the changes tended to weaken with the extension of durations in the plateau. Among obese populations, a higher F/B ratio was observed than that of normal individuals (31). Furthermore, a study has shown that the higher the F/B ratio of gut microbiota, the higher the maximal oxygen uptake and the higher the energy absorption and utilization capacity (32). In our study, the population showed a decreasing trend in F/B ratio after a short-term acute entry into the highland, which might lead to a weakened energy intake and utilization capacity and short-time weight loss during the initial period after entering the highland. With the time increasing in the plateau, adaptive changes would occur, and F/B ratios had a trend of recovery. A previous study has found that the F/B ratio in long-time residents at high altitude increased compared with those at low altitude (33), suggesting that long-term exposure to high altitude induces adaptive changes in the composition of gut microbiota, enabling the hosts to better adapt to the environment and live accordingly.

The functional clustering of hypoxia-responsive taxa includes opportunistic pathogens associated with mucosal disruption, divergent hypoxia responses of SCFA producers, and a decrease in psychobiotics linked to mental health. First, an increase exists in the relative abundance of opportunistic pathogens such as *Escherichia/Shigella* (kruskal.test), *Oscillibacter*, and *Ruminococcus*. As one of the lipopolysaccharide-producing bacteria, *Escherichia/Shigella* is a key bacterium in intestinal inflammation, with a significant increase in relative abundance in hosts with colitis, which was positively correlated with the degree of inflammatory cytokines and activity (34, 35). Studies found that the increasing abundance of *Oscillibacter* had a positive correlation with inflammatory cytokines like IL-6 and INF-γ in mice suffering from enterocolitis (36, 37), indicating *Oscillibacter* may serve as a potential gut inflammation marker at high altitudes. Notably, *Ruminococcus* has also been linked to irritable bowel syndrome and Crohn’s disease due to its ability to increase serotonin synthesis and produce inflammatory polysaccharides, which can trigger inflammatory factors like IFN-γ (38, 39). However, a higher concentration of *Ruminococcus* has often been found in the gut microbiomes of high-altitude healthy animals and humans and is positively related to SCFAs, suggesting potential benefits in acclimatization to hypoxic stress (8, 40, 41). Emerging evidence suggests that the pathophysiological effects of *Ruminococcus* may vary among different strains (42). The role of specific strains requires further research to be elucidated.

Second, SCFA-producing taxa exhibit dichotomous responses to hypoxic conditions at high altitude, forming two functionally distinct groups. The first group, composed of the hypoxia-responsive cluster, includes *Gemmiger* and *Parabacteroides*. As a butyrogenic member of the Firmicutes, *Gemmiger* exerts anti-inflammatory effects through butyrate biosynthesis (43). Clinical observations reveal that diminished *Gemmiger* abundance correlates with reduced SCFA levels and compromised immunoregulatory capacity, which may increase the risk of inflammatory responses similar to those in inflammatory bowel disease (44, 45). Our research demonstrated a temporary increase in *Gemmiger* abundance during acute high-altitude exposure, indicating that it may play a role in maintaining intestinal mucosal homeostasis and countering hypobaric hypoxia-induced inflammation. Similarly, after high-altitude exposure, our analysis showed a significant increase in *Parabacteroides* relative abundance. This result is consistent with previous epidemiological research indicating that high-altitude Han Chinese populations have higher baseline levels of this genus compared to sea-level populations (46). *Parabacteroides* has demonstrated beneficial functionalities in gut protection, including modulating intestinal immunity through downregulating pro-inflammatory mediators like TNF-α and IFN-γ, metabolically synthesizing SCFAs to supply energy substrates to colonocytes, and reinforcing epithelial barrier integrity (7, 23, 47). The concurrent increase of these genera suggests that they may work together metabolically and immunologically to cope with environmental stress. On the other hand, we identified a group of hypoxia-sensitive taxa, including *Faecalibacterium*, *Prevotella*, *Bifidobacterium*, and *Roseburia,* whose relative abundances were significantly reduced under hypoxic conditions. These taxa hold physiological importance, including maintaining epithelial integrity, regulating immune homeostasis, and supporting metabolic functions, as key producers of SCFAs such as butyrate (48, 49). The sustained depletion of these microbial communities may critically exacerbate intestinal barrier dysfunction through impaired colonocyte energy metabolism and diminished mucosal repair capacity due to SCFA deficiency. This pattern is similar to what is observed in intestinal inflammatory diseases, where the reduction of these taxa is related to the severity of the disease (44, 50, 51), suggesting hypoxia-induced microbiota alterations may share pathogenic pathways with chronic gut disorders. In detail, a reduction in *Faecalibacterium* abundance under acute hypoxia has been mechanistically linked to intensified intestinal inflammation and gastrointestinal symptom exacerbation (7, 52). Additionally, the abundance of *Prevotella* was inversely correlated with altitude across human populations—a pattern evidenced by Lan et al.’s (40) comparative study of Tibetan gut microbiota across 2,800–4,500 m elevations. This signature appears conserved across species, as parallel decreases occur in both human and animal models under hypobaric hypoxia (8, 33). Our analysis also highlights that *Prevotella* levels decline at higher altitudes, suggesting it may serve as a hypoxia signature of significant changes in gut microbiota under low-pressure, low-oxygen conditions. *Bifidobacterium* is crucial for maintaining the balance of the intestinal microbiota, protecting the mucosa, and ensuring barrier integrity in both infants and adults (12, 53). Studies have shown that when the altitude exceeds 5,000 m, a significant decrease in *Bifidobacterium* abundance can lead to potential gastrointestinal barrier damage and associated clinical symptoms such as nausea and vomiting (54). In addition, a reduction in *Roseburia* abundance, which is associated with various metabolic and intestinal pathologies (55, 56), implies that hypoxia may increase the risk of systemic diseases through microbial ecological disruption. The collective suppression of functionally convergent taxa highlights the gut microbiota’s susceptibility to hypoxic stress in high-altitude environments. Therefore, preventive management is necessary for individuals entering high-altitude areas to prevent significant gut microbiota disruptions that may lead to altitude diseases.

Third, the hypoxia-induced dysbiosis also has implications for mental health. There is an established association between reduced *Alistipes* abundance and depressive disorders (57), and a study has reported lower *Blautia* levels in patients with major depressive disorder (58). These findings acquire heightened relevance in high-altitude contexts where populations demonstrate elevated depressive symptom scores alongside gut microbiota alterations (59). The evidence mentioned above may indicate that the decrease in *Blautia* and *Alistipes* may indicate a connection between microbial ecology and neural pathways under hypoxic conditions. This positions specific microbial taxa as potential biological mediators linking high-altitude oxygen tension to physical and mental health outcomes, underscoring the necessity of microbiota-targeted interventions in high-altitude adaptations.

Metabolic reprogramming reflects a transition from crisis management to chronic adaptation. During acute high-altitude exposure, a notably enriched metabolic pathway is Xenobiotics Biodegradation and Metabolism, indicating that the gut microbiota can effectively degrade xenobiotic substances like drugs and pollutants. This suggests that at high altitudes, gut microbiota help maintain metabolic balance and enhance detoxification by processing potentially harmful substances. Aminoacyl-tRNA Biosynthesis pathway enrichment, linked to protein synthesis, suggests a need for enhanced protein production to support physiological regulation. Additionally, the enrichment of the Transcription Machinery pathway indicates improved gene expression regulation, allowing rapid responses to environmental changes. During prolonged adaptation to high altitudes, the enrichment of the Metabolism of Cofactors and Vitamins pathway highlights the gut microbiota’s ability to synthesize essential cofactors and vitamins to maintain nutritional balance and normal physiological functions. The Energy Metabolism pathway further illustrates the gut microbiota’s role in energy generation and utilization in low-oxygen conditions. The Bacterial Chemotaxis pathway showcases the bacteria’s ability to sense environmental signals, enhancing their adaptation to prolonged high-altitude exposure. Moreover, enrichment of the Chromosome-associated pathway points to improved genome stability and gene expression regulation within microbial communities, crucial for their health and functionality. Overall, this analysis reveals the dynamic changes in gut microbiota during both short-term and long-term high-altitude adaptations, highlighting the metabolic regulation and adaptation mechanisms to environmental challenges. This offers valuable insights into high-altitude physiology and microbial ecology.

The study had the following advantages. First, this study was a longitudinal cohort with a large sample size, which could more comprehensively and accurately investigate the changes in the composition and function of the gut microbiota in the population acutely exposed to high altitude, as well as the objective effects on the body, making up for the shortcomings of previous studies with small sample size. Second, existing evidence indicates that gut microbiota composition is influenced by multiple extrinsic factors, including dietary patterns, lifestyle habits, and ethnic variations (40, 46). Our study further confirms the role of lifestyle factors in shaping the composition of gut microbiota, which helps deepen the understanding of the relationship between lifestyle and gut health. It provides potential targets and a theoretical basis for improving health by intervening in gut microbiota through lifestyle adjustments. To minimize confounding effects highlighted in prior research, we implemented stringent experimental controls: exclusive enrollment of adult males to reduce gender-related variability; standardization of dietary intake across all study phases (pre-plateau, plateau acclimatization, and post-plateau return); longitudinal monitoring of altitude-associated physiological adaptations. Finally, this study investigated the effects of altitude on the gut microbiota and its function based on a real high-altitude setting, whereas most previously reported studies were either based on animal experiments or conducted in simulated plateau hypoxic conditions, such as in a hypobaric chamber. Therefore, our study more realistically reflects how hyperbaric hypoxia and low temperatures affect and reshape the structure and function of the gut microbiota in humans, as well as help people adapt to changes in altitude.

However, there are some limitations in this study. First, because of the limited research conditions, the samples collected for comparison (Group I) in this study were collected at an altitude of 800 m, which failed to obtain samples from the same population at sea level and failed to maximize the effect of high altitude on the gut microbiota. However, the fecal samples in this study came from the same population, and the effects of diets and other confounders were strictly controlled to minimize study heterogeneity. Second, the present study mainly analyzed the characteristics of gut microbiota in a large sample of people acutely exposed to high altitude but did not compare the differences with those in a cohort of indigenous inhabitants. Future studies should include indigenous high-altitude populations to disentangle genetic vs environmental effects on microbiota. Finally, this study reported the effects of the overall high-altitude factors on gut microbiota and did not subdivide the effects of various environmental factors, such as hypoxia, temperature, and ultraviolet light, on the structure and function of the bacteria. Further detailed studies were warranted to identify the environmental factors affecting the structure and function of the gut microbiota and to search for a targeted strategy for the prevention and treatment of acute highland illness.

### Conclusion

In summary, acute high-altitude exposure caused dramatic changes in gut microbiota, while prolonged exposure led to structural and functional reshaping. Our findings demonstrate that acute hypoxic exposure induces significant microbial ecological shifts, characterized by the increasing relative abundance of opportunistic pathogens (e.g., *Ruminococcus* and *Oscillibacter*) and bidirectional modulation of SCFA-producing bacteria—marked by increased abundance of *Gemmiger* and *Parabacteroides* alongside decreased levels of *Faecalibacterium*, *Roseburia*, and *Bifidobacterium*. While partial recovery of certain taxa was observed upon return to low-altitude regions, persistent structural remodeling of the gut microbiota induced by high-altitude conditions remained evident. At the functional level, the effects of highland exposure were mainly on reprogramming of metabolism-related pathways, such as the energy metabolism pathway, suggesting that low-pressure, low-oxygen environments facilitate host acclimatization through reshaping microbial structure and function. These findings enhance our understanding of how high-altitude environments reshape gut microbiota. To our knowledge, this is the first large-scale longitudinal study to track dynamic gut microbiota adaptation in humans during altitude transitions, with strict control of diet and lifestyle confounders.

## Data Availability

The original contributions presented in the study are included in the article/supplementary material. Further inquiries can be directed to the corresponding authors. The sequencing data have been uploaded to GenBank (BioProject PRJNA1194026).

## SUPPLEMENTAL MATERIAL

Supplemental material spectrum.02916-24-s0001.docx Fig. S1 and S2.

ASM does not own the copyrights to Supplemental Material that may be linked to, or accessed through, an article. The authors have granted ASM a non-exclusive, world-wide license to publish the Supplemental Material files. Please contact the corresponding author directly for reuse.
