# Supplementary material for: Human gut microbiota adaptation to high-altitude exposure: longitudinal analysis over acute and prolonged periods
Source: Microbiol Spectr. 2025 Apr 21;13(6):e02916-24. doi: 10.1128/spectrum.02916-24 (PMC12131729; doi:10.1128/spectrum.02916-24)
Supplement: Supplemental material — Fig. S1 and S2. [file spectrum.02916-24-s0001.docx]

**Human Gut Microbiota Adaptation to High-Altitude Exposure: Longitudinal Analysis Over Acute and Prolonged Periods**

**Runing Title: Gut Microbiota Adaptation to High-Altitude Exposure**

Xianzong Ma ^1,2^, Changwei Duan ^3^, Xiaoying Wang ^2^, Yurong Tao ^1, 2^, Lang Yang ^1,2^ ,Yongsheng Teng ^4^, Yuanming Pan ^5^, Mingjie Zhang ^2^, Junfeng Xu ^1^, Jianqiu Sheng ^1,^ ^2, 3, *^, Xin Wang ^2, *^, Peng Jin ^1, 2, 3, *^

^1^ Senior Department of Gastroenterology, The First Medical Center of Chinese PLA General Hospital, Beijing, 100853, China.

^2^ Department of Gastroenterology, The Seventh Medical Center of Chinese PLA General Hospital, Beijing, 100700, China.

^3^ Medical School of Chinese PLA, Beijing,100853, China.

^4^ Department of Gastroenterology, Chongqing General Hospital, Chongqing University, Chongqing 401147, China.

^5^ Cancer Research Center, Beijing Chest Hospital, Capital Medical University. Beijing, 101100, China.

Xianzong Ma, Changwei Duan, and Xiaoying Wang contributed equally to this article

***Corresponding author:**

Professor Peng Jin, Senior Department of Gastroenterology, The First Medical Center of Chinese NPLA General Hospital, No.28 Fuxing Road, Beijing, 100853, China. Tel: +86-10-66721299; Fax: +86-10-66721024, Email: jinpeng@301hospital.com.cn.

Professor Xin Wang, Department of Gastroenterology, The Seventh Medical Center of Chinese PLA General Hospital, No.5 Nanmencang, Beijing,100700, China. Tel: +86-10-66721299; Fax: +86-10-66721024, Email: chan805126@126.com

Professor Jianqiu Sheng, Department of Gastroenterology, The Seventh Medical Center of Chinese PLA General Hospital, No.5 Nanmencang, Beijing,100700, China. Tel: +86-10-66721299; Fax: +86-10-66721024, Email: [shengjianqiu@301hospital.com.cn](mailto:shengjianqiu@301hospital.com.cn).


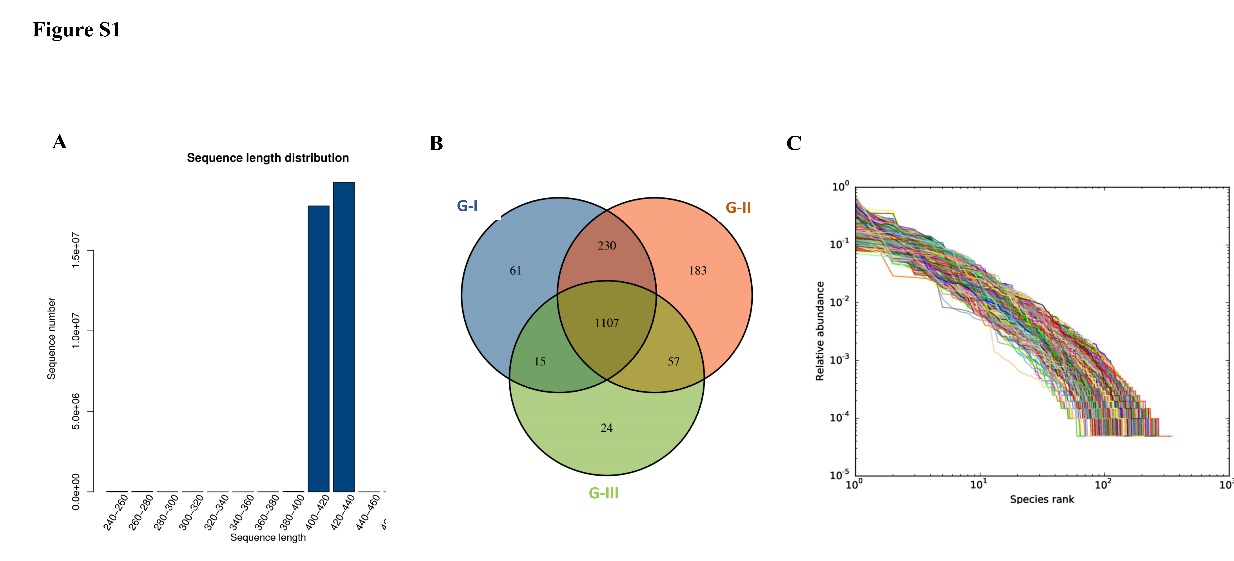


**Figure S1. Sequence information.** A. Sequence length distribution. B. Venn diagram illustrates the overlap of OTUs and unique OTUs in the gut microbiota among the three groups. C. Rank abundance curve. The X-axis represents the Rank of OTUs abundance, and the Y-axis represents the corresponding OTUs Abundance. The Rank-Abundance curve can intuitively reflect the classified abundance and evenness contained in the sample, that is, in the horizontal direction, the higher the value of the curve on the horizontal axis, the higher the abundance. In the vertical direction, the flatter the curve, the more uniform the species distribution.


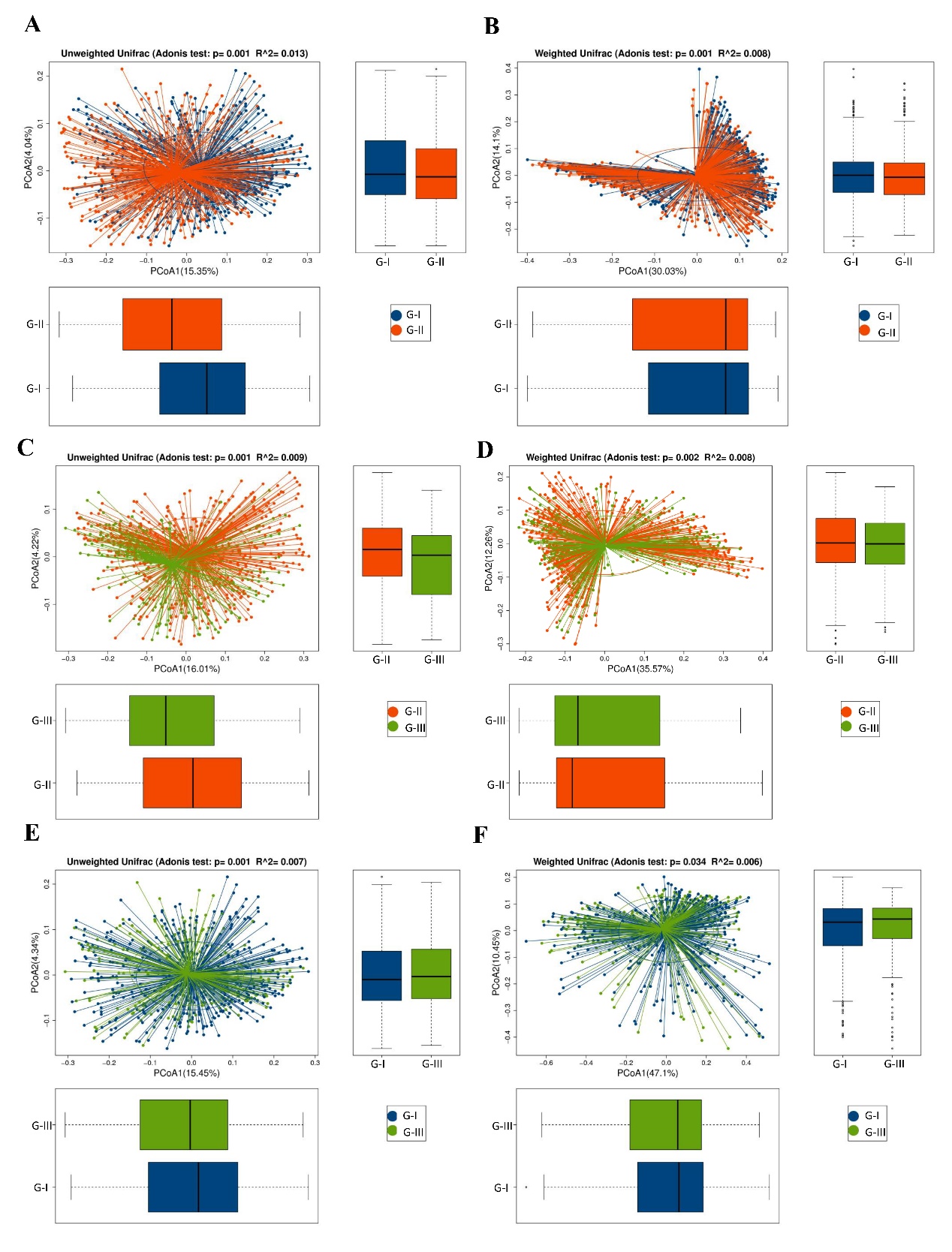
**Figure S2. Microbial community structure between three groups.** A, C and E: Unweighted Unifrac Principal component analysis (PCoA). B, D and F: PCoA of Weighted Unifrac. The percentage represents the contribution rate of the principal dimension to the sample difference.
